# Harnessing artificial intelligence for antimicrobial discovery and optimization

**DOI:** 10.1016/j.mib.2026.102724

**Published:** 2026-02-25

**Authors:** Ashley E Clements, Maureen R Fieldhouse, Allison S Walker

**Affiliations:** 1 Department of Chemistry, Center for Structural Biology, Vanderbilt Institute of Chemical Biology, Vanderbilt University, Nashville, TN, USA; 2 Department of Biological Sciences, Vanderbilt University, Nashville, TN, USA; 3 Department of Pathology, Microbiology, and Immunology, Vanderbilt University Medical Center, 1211 Medical Center Drive, Nashville, TN 37232, USA

## Abstract

The rise of antimicrobial-resistant pathogens has outpaced the traditional methods of drug discovery and development, emphasizing a need for new and innovative approaches to identifying novel antibiotics. Artificial intelligence (AI) poses new opportunities to overcome the challenges in traditional drug discovery by accelerating the identification, design, and optimization of bioactive small molecules and antimicrobial peptides. AI-driven genome mining allows for the identification and prioritization of biosynthetic gene clusters, while advanced AI models facilitate molecular property prediction, predicted binding interactions, and novel structure design. This review explores the advancements that AI has enabled in antimicrobial discovery and design, as well as its current limitations.

## Introduction

Over the past century, modern medicine has heavily relied on antimicrobials to reduce infections and mortality caused by microbial pathogens, including bacteria, viruses, parasites, and fungi. Resistant bacteria are of especially high concern; therefore, we will focus this review on antibacterial discovery. In 2017, the World Health Organization (WHO) identified six priority bacterial pathogen targets for antimicrobial development, *Enterococcus faecium, Staphylococcus aureus, Klebsiella pneumoniae, Acinetobacter baumannii, Pseudomonas aeruginosa and Enterobacter* spp., known as the ESKAPE pathogens [1]. The widespread use of antibiotics has led to the development of drug-resistant bacteria that render current pharmaceuticals ineffective. Antibiotic resistance can arise through random genetic mutations or horizontal gene transfer, and generally functions through changes in the antibiotic’s target or target pathway, decreased antibiotic accumulation in the cell through decreased entry or increased export, or antibiotic degradation [2,3]. The 2025 Global Antibiotic Resistance Surveillance Report, generated by WHO, estimated that approximately one in six laboratory-confirmed bacterial infections worldwide were caused by antibiotic-resistant bacteria. From the same report, 104 countries reported antimicrobial resistance impacting the healthcare system, highlighting the pressing need for antibiotics [4].

**Figure 1:**
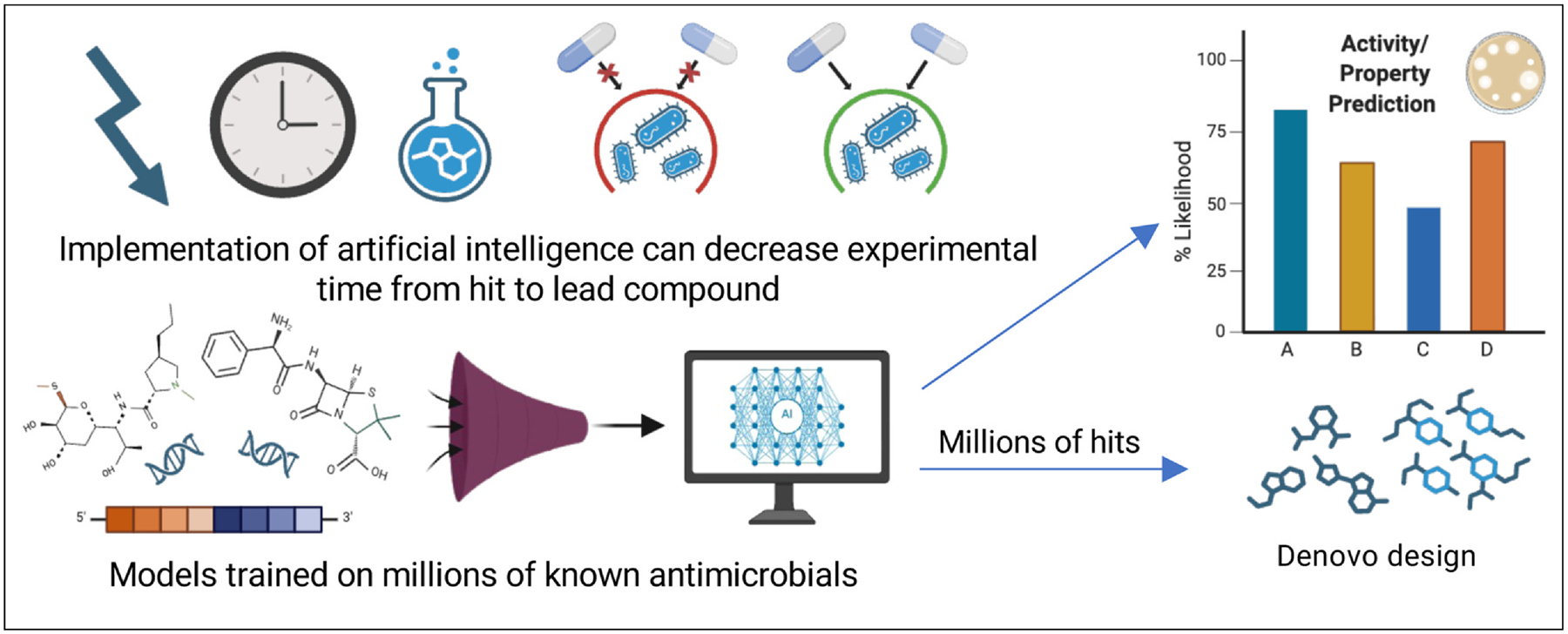
Overview of harnessing artificial intelligence for antimicrobial discovery and optimization. Created using BioRender.

New compounds, particularly those with novel mechanisms of action (MoA), are needed to address antimicrobial resistance. Resistance to any new antibiotics will likely emerge quickly [1], and therefore, the pace of antimicrobial discovery must be accelerated. Traditional methods of antimicrobial discovery, for both natural and synthetic compounds, are time-consuming. Major bottlenecks in natural product discovery include the accidental rediscovery of known compounds, labor-intensive extractions, and bioactivity screening [5]. Discovery of synthetic antibiotics is limited by low hit rates in high-throughput screens (HTS) [6]. Time and resource intensity are significant hurdles in the development of antibiotic therapeutics, with the average time from discovery to market taking approximately 12 years. Artificial intelligence (AI) is currently used in the early stages of the antibiotic discovery process but has shown reduced discovery and development times through rapid screening of chemical libraries, property prediction, *de novo* structure design, and target identification (Figure 1) [7]. Here, we review some recent advances in this area as well as remaining challenges.

## Small molecule antibiotic discovery

### AI in genome mining

AI tools have the potential to overcome the bottlenecks inherent in traditional ‘trial and error’ approaches in natural product discovery. This begins with the use of AI in genome mining to identify potential biosynthetic machinery and prioritize secondary metabolite producers based on predicted molecular features and activity.

Genome mining is the process of analyzing genetic data to identify biosynthetic gene clusters (BGCs), which encode the machinery that produces secondary metabolites. The most widely used tool for BGC detection and characterization, antiSMASH, uses curated profile-Hidden Markov Models (pHMM) in combination with a rule-based system to identify BGCs [8]. However, these HMM-based algorithms often struggle with capturing higher-order dependencies between genes, limiting their accuracy and generalizability to new BGC classes.

Models like DeepBGC, BiGCARP, and BGC-Prophet use deep learning to overcome limitations of traditional rule-based approaches, the specifics of which are summarized in Table 1 [9–11]. These models aim to improve BGC identification and characterization but generally have higher false-positive rates than stricter rule-based methods while relying on training sets of known BGCs with protein domain features, limiting their ability to identify truly novel classes of BGCs. Both DeepBGC and BiGCARP perform well for classification of BGCs, with area under receiver operating characteristic curves of 0.921 and 0.941, respectively, for domain-level classification performance [9,10]. Of the two, only DeepBGC has the capacity to classify BGCs by their predicted activity and is limited by a small data set, resulting in low predictive accuracy, with an average area under receiver operating characteristic curve of 0.603 and precision of 0.536 when classifying BGCs by underlying activity [9]. While many of the models discussed prioritize the discovery of novel BGC classes, they do pose the risk of false novelty detection and rediscovery of cryptic variants of known BGCs. Users should be aware of these limitations when utilizing AI for genome mining, specifically when prioritizing the discovery of unique BGCs and metabolites.

The model developed by Walker & Clardy in 2021 [12] predicts the likelihood of secondary metabolite activity, including antibacterial activity, using a bioinformatics-based AI approach. It classifies BGCs identified by antiSMASH as active or inactive based on protein domain and self-resistance features. As shown by Ancajas et al. [13], the model has successfully predicted antibacterial activity, demonstrating strong potential for activity-based prioritization of BGCs and bacterial producers. Similarly, the PRediction Informatics for Secondary Metabolomes system by Skinnider et al. [14] uses numerous HMMs to classify BGCs and virtual reactions to predict metabolite structures, from which activity is inferred. However, PRediction Informatics for Secondary Metabolome’s rule-based framework limits its ability to generalize to novel or uncharacterized BGC families.

The identification of antibiotic resistance genes (ARGs) is another key strategy for secondary metabolite discovery. Organisms use ARGs as a means of protection against the antibiotics they produce, so their presence within or adjacent to BGCs can provide insight into their MoA. Tools like Resistance Gene Identifier [15,17] as part of the Comprehensive Antibiotic Resistance Database (CARD), and Antibiotic Resistance Target Seeker (ARTS) [16] utilize traditional bioinformatic target-directed genome mining to flag strains and BGCs as putative antibiotic producers by detecting possible resistant housekeeping genes. There are also AI-based methods for the identification of ARGs, including DRAMMA [17], HMD-ARG [18], ARG-SHINE [19], and DeepARG [20]. For readers who want a more in-depth explanation of ARG identification, see the review by Bilal et al. [21].

### Property prediction and design

AI-based activity prediction, using molecular finger-prints, graph-based models, descriptor-based representations, and chemical language models (CLMs), has streamlined the antibiotic discovery process by prioritizing compounds from large chemical libraries. These models reduce screening time by filtering out compounds with lower chances of being active and improving hit rates in HTS.

Molecular fingerprints represent compounds as a vector that encodes the presence or absence of specific molecular features or substructures within a compound. Fingerprints can be used for tasks such as similarity searching, quantitative structure–activity relationship (QSAR) modeling, and virtual screening [22]. In contrast, graph neural networks (GNNs) convert compounds to a graphical representation where nodes correspond to atoms and edges correspond to chemical bonds, maintaining more detailed information and allowing AI models to learn about the most important features of the molecule [23].

Resources like Chemprop [23] and FinGAT [24] utilize these different representations to predict the chemical properties of small molecules. Chemprop takes SMILES string inputs and converts them into molecular graphs and then uses a message passing neural network, a specific type of graph convolutional neural network, to predict the molecular properties. FinGAT combines 2D fingerprints and a graph attention network to predict antibiotic activity classification. Additionally, narrow chemical space coverage can lead to model development of ‘local chemical intuition’ as opposed to generalizable predictive power. This limits the out-of-domain inferences that can be made by these models, notably for natural products and highly decorated scaffolds, unusual ionization states, tautomer, and stereochemical patterns. The variability in experimental labels, particularly for biochemical assays (IC50s, EC50s, minimum inhibitory concentrations, etc.) and ADMET endpoints, can lead to the models becoming overfit to the noise rather than the mechanistic signal. Data leakage, for example, inclusion of the same or similar data in the training and test data, can make these overfit models appear to perform more accurately than they will in practice. This also limits the dynamic range of classification due to censoring at the assay limits/endpoints. Furthermore, specialized domains (polymeric repeating units, peptide and oligonucleotide backbones, 3D conformers with stereo-electronic effects) often require representation beyond standard SMILES graphs, which Chemprop’s default featurization cannot encode, ultimately restricting performance for complex structure patterns [25]. There have been promising results from these models, Chemprop in particular, that have yielded the identification of antibiotic compounds effective against species prone to resistance [26–31]. Notable examples of AI hit discoveries include halicin and enterololin, utilizing trained deep neural networks for HTS and generative docking for property prediction, respectively [28,31]. Cross-study comparison of many of these models is not feasible as *in vitro* validation conditions (strains, assay format, minimum inhibitory concentration thresholds, etc) vary between studies. Standardization of experimental validation conditions would greatly improve the interpretability of *in vitro* testing of these predictive models.

Recently, interest has grown in utilizing CLMs for property prediction and structure design. CLMs use deep learning, adapted from natural language processing, to analyze and generate molecular structures represented as strings like SMILES. These models are pretrained on vast unlabeled chemical data and then fine-tuned for particular tasks, a process that applies to both CLMs and GNNs [32,33]. CLMs include Effichem [34], Molformer [35], and ChemBERTa [36] and have been used to generate antibiotics with activity in the low micromolar range [37]. There are several limitations with CLMs, particularly with the computational cost of pretraining and fine-tuning [38]. Additionally, the use of SMILES representation can result in sensitivity issues, particularly when varying the canonicalization process can result in varying prediction outcomes. There are several approaches that are currently being investigated to address these concerns, including token deletion, atom masking, and bioisosteric substitution [39]. For a more comprehensive review of the discovery of novel antibacterial small molecules, see the reviews by Cardona et al. [40] and Arnold et al. [41].

Generative AI enables the *de novo* structural design of antibiotics. AutoMolDesigner [42] combines deep learning–enabled molecular generation and automated machine learning (ML)-based antibacterial activity prediction. This tool generates libraries of small-molecule antibiotics and then predicts their activity and molecular properties. Other platforms have used genAI to create libraries of thousands of proposed antimicrobial compounds, some of which have been experimentally determined to be active against *Neisseria gonorrhoeae* and *Staphylococcus aureus* and other multidrug-resistant pathogens [43–45]. Generative models are useful in proposing chemically novel structures, but unconstrained models often prioritize over-decorated scaffolds, chemically unstable motifs, and infeasible stereochemical patterns. Without synthetic accessibility filters and constraints, the models propose molecules that score well on computational objectives but are either impractical or impossible to synthesize. While the use of generative AI is widespread, some unexpected outcomes, such as model collapse and reward hacking, can occur. During development, it is important to note that generative models can sometimes limit the chemical diversity of their outputs, even outputting similar compounds repeatedly, and mimicking the original training set. For more information on generative drug design and limitations, see the reviews by Sherman et al. [46], Xie et al. [47], and Yoshizawa [48].

### Target identification

Progression from hit to drug candidate is often hindered by a lack of understanding of its mechanism [50]. AI models can be used to predict drug-protein interactions, potentially narrowing down the on- and off-target proteins that interact with the hit [51]. Najm et al. utilize both support vector machine and random forest chemogenomic models to predict drug-target interactions (DTIs) by mapping interactions between small molecules and proteins [52]. Explainable AI (XAI) has also gained prominence in the antimicrobial and drug discovery landscape, allowing researchers to identify key substructures or interactions and use those to guide optimization later in the design/discovery process. XAI unites predictive accuracy with both transparency and interpretability, which can be imperative in medicinal research such as DTIs. For more information about how XAI can be incorporated into the drug discovery pipeline, readers should refer to this comprehensive review [53]. To minimize the probability of incorrect predictions due to overconfidence, evidential deep learning can be used to quantify uncertainty in DTI predictive models [51]. AI-assisted protein structure prediction methods, such as AlphaFold [54] or RoseTTAFold [55], can be used as starting points for traditional docking, can be integrated into AI-based docking methods such as DiffDock-L [49], or can be used for co-folding with the ligand if they support ligand structures. For a comprehensive review highlighting the successes and pitfalls of target identification using ML methods, readers are prompted to read the following review [56]. Specifically focused on combating antibiotic resistance, Ferrero et al. [50] leveraged Open Targets with a semi-supervised approach to harness gene-disease association data and predict potential drug targets, broadening the spectrum of investigational antibiotics. In combination with structure-based design, this could simplify the small-molecule antibiotic design process and minimize the likelihood of off-target effects.

Some major pitfalls of target identification and the incorporation of AI result from their training datasets. Many datasets remain unbalanced, with varying ratios of positive and negative compounds in the training set [57]. However, recent improvements to larger labeled datasets could overcome this bottleneck. Researchers could also augment the data to increase the size and improve the balance of the dataset, for example, by using synthetic negative sampling, which creates artificial false examples. Another current limitation that might be overcome by time is the lack of structural data for key protein targets. The number of sequenced proteins greatly outnumbers the number of solved crystal structures. With recent advancements using cryogenic-electron microscopy [58] and improvements in AI-predictions of protein structure [54], the number of quality structures to screen against will increase over time. These models are key in expediting the time to identify potential targets, but the ability to validate these predictions experimentally, through strategies such as knock-outs and chemoproteomics, still presents a significant bottleneck in the target identification process.

## AI-driven discovery and design of antimicrobial peptides

Antimicrobial peptides (AMPs) are a promising alternative to small-molecule drugs due to their broad-spectrum activity and unique mechanisms of action, which reduce the likelihood of resistance [59,60]. Most AMPs are amphiphilic, ranging in length from 6 to 100 amino acids, and act as defense mechanisms for the producer from pathogens.

### Mechanism of action and limitations

AMPs have varying mechanisms of action. While most AMPs act by disrupting the microbial membrane or inhibiting the ribosome, others can target DNA replication, transcription, and translation-related proteins [61]. Although their MOA is effective against many pathogens, their *in vivo* effects can include cytotoxicity and increased off-target effects when compared to other known antibiotics. Disruption of the membrane or the development of micelle-like AMPs that inhibit biofilm activity are mechanisms that are less prone to developing resistance [62]. A peptide’s ability to bind to the negatively charged surface of the bacterial cell membrane allows for translocation across the membrane through both electrostatic and hydrophobic interactions [59]. Numerous AMPs have been identified in bacteria, fungi, plants, and even the human gut microbiome [63]. While AMP discovery has been primarily experimentally driven, the recent creation of AMP-annotated databases combined with fast peptide synthesis and testing has propelled computationally aided discovery and design of AMPs [59].

### Classifier models

Early work on AI AMP identification employed classifier models [60,64]. Training data, including peptide sequences and associated properties, were drawn from annotated databases, such as APD3. The models are then trained to predict antimicrobial activity from peptide sequences. The best candidates are selected or classified based on criteria set by the user [65], such as predicted minimum inhibitory concentration or probability of activity.

The earliest computational models for classifying AMPs emerged in the early 2000s, when the Hancock group at the University of British Columbia employed QSAR modeling to associate peptide structural features with biological activity [59]. Because of their relatively short length and low sequence similarity, traditional linear models fail to capture the antimicrobial activity of AMPs. To overcome this limitation, Hancock et al. used atomic-resolution chemical descriptors as inputs to an artificial neural network to enhance predictive performance [59]. In addition to classifiers, there are also generative models capable of designing AMPs [66].

### Genome mining

Similar to small natural product discovery, genome mining can be applied to peptides, informing the discovery and design of novel AMPs [67]. Recent approaches for AMP discovery have led scientists to turn inward, focusing on the human gut microbiome. In 2022, Ma et al. employed deep learning algorithms to analyze vast genomic datasets from the gut microbiome and identify possible peptides with high antibacterial potency [63]. Unlike previous approaches, five neural network models, including LSTM, ATT, and BERT, were combined to generate highly accurate genome mining hits. This pipeline surpassed other currently available AMP ML models trained using the same dataset, based on the Area Under the Precision-Recall Curve metric, with the highest value of 0.9244 out of 1. Subsequent synthesis of predicted AMPs mined from the microbiome and assays determined the model’s true positive rate to be 83.8% [63].

Tackling the same problem but with a different approach, the de la Fuente lab developed an antibiotic peptide de-extinction model. Through paleoproteome mining and ML, they identified encrypted peptides, peptide fragments within proteins, which had antimicrobial activity from both modern humans and our evolutionary ancestors. In their 2024 paper, they created a data set of over 10 million peptides, predicting 37,176 with antimicrobial activity. Sixty-nine of these peptides were synthesized, and 41 were found to be active, validating the model [68]. Outside of their de-extinction model, the de la Fuente lab has also employed deep learning approaches for AMP discovery in the archaeal proteome, global venom, and microbiome datasets [69–71].

Leveraging the idea that *in silico* evolution can expedite experimental bench work, Wang et al. set out to develop an explainable deep learning and virtual evolutionary model to predict AMPs against human pathogens. The pivotal feature of their model is the explainability feature, which allowed them to develop an algorithm for the virtual directed evolution of AMPs with enhanced antimicrobial potency. After analysis of only 38 genomes, 32 predicted AMPs eliminated over 95% of the pathogenic bacterial load *in vivo* [72]. For more details, readers should refer to previous reviews on the AI development of AMPs [41,73–75].

### Optimization

While AMPs are relatively small, they pose some challenges regarding cytotoxicity, off-target effects, stability, and immunogenicity. AMP optimization ML models have been developed to counteract this. HydrAMP is the first model that directly optimizes generated sequences iteratively while satisfying antimicrobial activity conditions predicted by previous classifier models. HydrAMP is an extension of a conditional variational autoencoder (cVAE), leveraging two functionalities: generating analogs of existing peptides with specified antimicrobial properties (analog generation) and generating peptides *de novo* (unconstrained generation) [60]. Drawbacks of this model include its training on only *E. coli* inhibition and neglect of host toxicity. Previous models have considered activity against both Gram-positive and Gram-negative pathogenic bacteria, but lacked a *de novo* design feature. Models in this category utilize a combination of classifier models and iterative redesign, using known AMPs as a template [60].

### Ribosomally synthesized and post-translationally modified peptides

Ribosomally synthesized and post-translationally modified peptides (RiPPs) are a diverse group of secondary metabolites that have a long history of antimicrobial activity. Many RiPPs contain modifications that improve their protease resistance [76], addressing some of the drawbacks of linear AMPs. Lasso peptides, characterized by a unique structural feature in the shape of a lasso, and lanthipeptides, characterized by lanthionine-containing cycles, have both been shown to have potent activity against pathogens [77]. Recently, computational methods, including AI models, have been developed to model peptides as well as predict their properties. In 2025, Tydings et al. developed the first structural modeling software for lantipeptides, which enables modeling of lanthipeptides interacting with their target and optimization using the Rosetta design protocol [78]. Other structural models, including LassoESM and LassoPred, leverage Evolutionary Scale Modeling and classifiers to predict the enzyme compatibility and activity or structure of lasso peptides, respectively, with relatively high accuracy [79,80]. Further details of each model are summarized in Table 2. However, additional work is still needed to expand these advances to other RiPP classes and to increase the throughput of RiPP expression and discovery to obtain data for further improving these methods. Researchers commonly face challenges with RIPP expression and scalability, lengthening the process from hit to lead compound. The use of models predicting peptide activity and structure could aid in prioritizing candidates and reducing experimental time.

## Challenges and limitations

Despite the numerous advantages of utilizing AI in the discovery and development of novel antibiotics, there are several limitations of AI. The main challenge of using AI in drug discovery is the lack of high-quality, annotated, and standardized data. These models require vast amounts of high-quality data to produce consistent and reliable results. Models trained on small quantities of data are more likely to be overfit and are less able to generalize [82]. In the case of LassoPred, Ouyang et al. highlight that their model was only able to be trained on 47 solved crystal structures, which are a necessary component of any structure-based model [79]. Issues like this are common, and the only way to improve the diversity of the training sets is to obtain more experimental data. Even when large quantities of data are available, they are often amassed from a variety of sources and are therefore rarely standardized. Additionally, biological systems are complex and sensitive to assay conditions that may not be standardized. To predict accurate protein–drug interactions, the algorithm must be able to navigate this complexity.

One of the largest concerns with AI in drug discovery and design is the interpretability, generalizability, and reliability of the model. There are several models available that have a bias towards a certain subset of output, for example, Gram-negative or Gram-positive bacteria, making them inefficient and unreliable for broader spectrum application. The datasets used to train some AI models also do not include activity against multidrug-resistant strains, which creates challenges when applying novel antibiotics in the clinic. Another limitation in the field is the lack of standardization across different models predicting antimicrobial properties. This is due to a myriad of reasons, including a lack of experimental testing and benchmark standardization across different AI models and institutions. Addressing these limitations would aid in the large-scale implementation of AI in the antibiotic discovery process, which in turn would help to combat the growing challenge of antimicrobial resistance.

## Conclusion

AI holds significant promise to fight the emerging threat of antibiotic resistance. AI has accelerated the discovery of antimicrobial natural and synthetic small molecules and larger peptides. Across both small molecules and AMPs, limitations exist with current AI models, including a lack of high-quality, standardized data and challenges in addressing model reliability and generalizability. When deciding between deep learning approaches and traditional rule—based models, we have found that deep learning approaches work best in situations of extracting complex, nonlinear patterns and generalizing across chemical space, while rule-based approaches excel at utilizing prior chemical knowledge and are better for transparency and interpretability. AI in drug discovery is still in its infancy, and additional application of the methods described in this review to real drug discovery pipelines is needed to determine which methods will be the most transformative. Additional advancements in high-throughput experimentation are likely needed to fully realize the promise of AI in antimicrobial discovery. Despite these limitations, AI presents novel opportunities to overcome the challenges faced by traditional drug discovery, accelerating the identification, design, and optimization of bioactive small molecules and AMPs.

## Figures and Tables

**Table 1:** Small molecule AI tools. Summary of the discussed AI models for antimicrobial small molecule discovery and design.

AI Tools	Methods	Applications	Refs.
DeepBGC	BiLSTM, RNN, and random forest classifiers	Classify BGCs by product class and molecular activity	[9]
BiGCARP	self-supervised masked language model	BGC class detection and identification	[10]
BGC-Prophet	transformer-based language models	Location-dependent gene relationship identification	[11]
Walker & Clardy	random forest with extra-randomized trees, support vector machine (SVM), and logistic regression	Predict the likelihood of secondary metabolite activity	[12]
PRISM	HMMs and RDKit	Classify BGCs and predict secondary metabolite structures from which activity can be inferred	[14]
Chemprop	message passing neural networks	Prediction of bioactivity given the structure of a compound	[23]
FinGAT	2D fingerprints and a graph attention network	Prediction of antibacterial activity given the structure	[24]
Effichem	transformer-based chemical language models (CLMs) in combination with domain-specific chemical features	Molecular property prediction and determination of substructures with associated activity	[34]
Molformer	CLM with linear attention mechanism	Prediction of chemical and quantum-mechanical properties	[35]
ChemBERTa	BERT-style transformer that learns molecular fingerprints through semi-supervised pretraining	Higher throughput chemical property prediction	[36]
AutoMolDesigner	RNN for focused library generation of small-molecule antibiotics and an automated machine learning (AutoML) framework	Molecular generation and antibacterial activity/property prediction	[42]
DiffDock-L	Diffusion and confidence models with ML-based docking methods	Molecular docking and drug-binding structure prediction	[49]
GNEprop	GNN equipped with an explainability pipeline	Identification of compounds with antibacterial activity and the motifs conferring activity. Additionally, characterizes structural uniqueness with respect to activity	[33]
Krishnan et al.	Fragment-based: GNNs for screening of fragments for activity then CReM and VAE *De novo*: chemically reasonable mutations (CReM) and a variational autoencoder (VAE) model	Fragment-based and *de novo* generation of antibacterial compounds	[44]
SyntheMol	Monte Carlo tree search (MCTS)	Generation of novel compounds with antibacterial activity	[45]

**Table 2:** AMP AI tools. Summary of the discussed AI models for antimicrobial peptide discovery and design.

AI tool	Methods	Applications	Refs
QSAR	Structure-activity models	Design of Small Peptide Antibiotics Effective against “Superbugs”	[59]
HydrAMP	Conditional, generative model, cVAE	Generation of novel peptide sequences satisfying given antimicrobial activity conditions	[60]
AMP identification pipeline from the human gut microbiome	LSTM, Attention, and BERT,	Identify possible peptides with high antibacterial potency from a large genomic database	[63]
WGAN-PG-designed peptides	WGAN-GP	Can identify novel AMP candidates from known AMPs	[81]
SMEP	Empirical selection, classification, ranking, and regression enhanced by an incremental learning mechanism	Can screen known peptide libraries to identify antimicrobial activity	[64]
ProteoGPT, AMPsorter, BioToxiPept, AMPGenix	Multiple LLMs compiled	Mining and generation of AMPS specifically against CRAB and MRSA	[66]
APEX	Multitask learning architecture, encoder neural network, FCNNs	Bacterial-strain-specific antimicrobial activity predictor. In this case, trained on ancient genomes	[68]
APEX 1.1	Multitask learning architecture, encoder neural network, FCNNs	Antimicrobial activity predictor retrained on updated data	[69]
Macrel	Random forests	Predicts AMPs from large peptide datasets with an emphasis on precision over recall	[71]
EvoGradient	Long Short-Term Memory (LSTM), Recurrent Neural Network (RNN), Attention, Bidirectional Encoder Representation from Transformers (BERT)	Predicts antimicrobial potency of peptides. After prediction, the model can perform in silico directed evolution to increase potency.	[72]
LassoPred	Classifier	A tool to predict the structure of lasso peptides	[79]
LassoESM	ESM-2 architecture, Protein language models	For lasso peptide property prediction and substrate compatibility	[80]

## Data Availability

No data were used for the research described in the article.
